# Unraveling the Relative Importance of Oral and Dermal Contaminant Exposure in Reptiles: Insights from Studies Using the Western Fence Lizard (*Sceloporus occidentalis*)

**DOI:** 10.1371/journal.pone.0099666

**Published:** 2014-06-18

**Authors:** Scott M. Weir, Larry G. Talent, Todd A. Anderson, Christopher J. Salice

**Affiliations:** 1 The Institute of Environmental and Human Health, Department of Environmental Toxicology, Texas Tech University, Lubbock, Texas, United States of America; 2 Department of Natural Resource Ecology and Management, Oklahoma State University, Stillwater, Oklahoma, United States of America; University of Tuscia, Italy

## Abstract

Despite widespread recognition of significant data deficiencies, reptiles remain a relatively understudied taxon in ecotoxicology. To conduct ecological risk assessments on reptiles frequently requires using surrogate taxa such as birds, but recent research suggests that reptiles have significantly different exposure profiles and toxicant sensitivity. We exposed western fence lizards, *Sceloporus occidentalis*, to the same quantities of three model chemicals via oral (gavage) and dermal (ventral skin application) exposure for either 24 or 48 hours. Three phthalate esters (di-methyl phthalate [DMP], di-iso-butyl phthalate [DIBP], and di-n-octyl phthalate [DNOP]) were chosen as model chemicals because they represent a gradient of lipophilicity but are otherwise structurally similar. Overall, the more lipophilic phthalates (DIBP and DNOP) were found to have higher concentrations in tissues than the less lipophilic DMP. Significant differences in tissue concentrations between DIBP and DNOP were tissue-dependent, suggesting that delivery to a site of action following exposure is not only a simple function of lipophilicity. In dermal treatments, DMP usually had fewer detections (except in ventral skin samples), suggesting that lipophilicity (log K_ow_>2) is a requirement for uptake across the skin. In general, tissue residues were greater in oral treatments than dermal treatments (significant in adipose and liver tissue), but differences were driven strongly by differences in DMP which did not appear to be absorbed well across skin. When differences in tissue residue concentrations between oral and dermal exposure did occur, the difference was not drastic. Taken together these results suggest that dermal exposure should be considered in risk assessments for reptilian receptors. Dermal exposure may be an especially important route for reptiles as their ectothermic physiology translates to lower energetic demands and dietary exposure compared to birds and mammals.

## Introduction

Reptiles appear to be declining globally, and contaminants are one of many possible stressors contributing to these declines [1]. Despite recognition that chemical contaminants can and do impact reptiles, the taxon remains understudied in ecotoxicology [2], [3] despite several calls for more research [4], [5]. One of the consequences of a lack of ecotoxicity and exposure data is that ecological risk assessments (ERA) on a reptilian species will have considerable uncertainty reducing the utility of assessment results for sound management decision-making. Because of a lack of reptile-specific data, birds are often used as surrogates for reptiles in ERAs. The use of avian surrogates in reptile ERAs, however, may be inappropriate and could result in gross underestimates of risk under certain exposure scenarios, or when reptiles are far more sensitive than birds to a particular contaminant [3]. In addition, there has been an increased interest in explicitly considering reptiles and amphibians in the risk assessment process (see, for example, [6]). Unfortunately, so few data on reptile ecotoxicity are available that even a generalized understanding of contaminant toxicity and exposure is elusive in this taxon.

The introduction of the western fence lizard (*Sceloporus occidentalis*) as a vetted model organism for reptile ecotoxicity studies [7] has played a large role in increasing the availability of reptile toxicity data in recent years for metals [8], pesticides [9], and compounds of military importance [10], [11]. However, both estimates of exposure and estimates of toxicity are needed to conduct meaningful ERAs. Experimental investigations of the dynamics and importance of contaminant exposure are rare for most terrestrial taxa [12] and, to our knowledge, no data on the nature and extent of contaminant exposure exist for any reptile species.

Current approaches in ERAs generally focus on dietary exposure as the sole (or dominant) route of contaminant exposure such that other routes are infrequently considered (e.g., [13]). There has been a recent surge in interest regarding the explicit incorporation of dermal exposure into ecological risk assessments [14], [15]. Dermal contaminant exposure may be more important than dietary exposure immediately following a pesticide spray, even in birds, which generally have very high dietary exposure as a result of high energetic demands [16], [17]. The relative importance of dermal exposure is likely to be especially significant for reptiles as their lower metabolic rate results in fewer daily feeding events compared with birds and mammals. Reptiles would, therefore, be expected to have less dietary exposure than birds and mammals of a similar trophic level. Importantly, because reptiles have a high percentage of body surface area potentially in contact with a contaminated substrate, the relative contribution of dermal exposure may be quite high [3].

The purpose of this research was twofold. The first objective was to determine the relative contribution of dermal and oral contaminant exposure in a model reptile, the western fence lizard, under controlled laboratory conditions. Secondly, we sought to determine the role of lipophilicity in understanding and potentially predicting the relative importance of oral and dermal exposure to total contaminant exposure. To address these objectives, we exposed western fence lizards to three phthalate esters representing a lipophilicity gradient via both oral and dermal routes. We discuss results in light of the need to improve ERAs for reptiles.

## Materials and Methods

### Ethics Statement

All experimental procedures were approved by the Institutional Animal Care and Use Committee at Texas Tech University (AUF 10062-10). Euthanasia methods followed the 2007 Euthanasia Guidelines of the American Veterinary Medical Association.

### Study Organisms

Male, adult, western fence lizards, *Sceloporus occidentalis*, were acquired from a colony maintained at Oklahoma State University. The founders of the colony were captured in the San Joaquin Valley, California [7]. Lizards were held individually in plastic containers measuring 11 cm deep×15.5 cm wide×28.5 cm long. Containers had 1 kg of a locally collected top soil placed on the bottom as substrate. Lizards were provided a small water dish (10 mL volume) for *ad libitum* drinking. Lizards were fed 2 large mealworms (*Tenebrio sp*. approximate weight = 0.15 g) every other day prior to initiation of experiments. The lizards were given a 14∶10 light dark cycle and a heat lamp was provided for 6 hrs each day for thermoregulation. The heat lamp created a gradient of approximately 26–34°C within the container when the lamps were on. When the lamps were off, temperatures were maintained at 23±2°C.

### Model Chemicals

Three phthalate esters were chosen as “model chemicals” representing a gradient of lipophilicity. Phthalates are useful model chemicals for exposure studies because they become more lipophilic as the length of the side chain increases; however, there is relatively little change in the overall structure of the chemical. This reduces the possible confounding factor of chemical structure on dermal and gastrointestinal absorption. Phthalate esters are also not acutely toxic even at high exposure levels, so exposure concentrations that will increase the probability of detection will not cause acute toxicity, which may affect metabolism and other physiological processes. The chosen phthalates with associated log K_ow_ values (summarized in [18]) were: di-methyl phthalate (DMP, log K_ow_ range: 1.4–1.9), di-iso-butyl phthalate (DIBP, log K_ow_ range: 4.11–4.27), and di-n-octyl phthalate (DNOP, log K_ow_ = 5.22–8.18). The log K_ow_ is a measure of lipophilicity and represents the ratio of a chemical in octanol and water following spiking and mixing. A higher log K_ow_ indicates greater liphophilicity.

We acquired pure phthalates from Chemservice (Westchester, PA, USA). The purified form of phthalates is liquid; therefore, our doses were applied without requiring a carrier solvent. To ensure that each lizard was given the same dose, we used a pipette to provide a 10 µL dose of each phthalate to each lizard. We maintained the same dosing volume across all three phthalates. Each phthalate had a different density so a 10 µL exposure solution resulted in different masses applied for each phthalate. The mass (mean ± SE) of 10 µL of each phthalate from 3 repeated measurements on a balance was: 8.71±0.02 mg for DMP, 9.56±0.17 mg for DIBP, and 11.19±0.52 mg for DNOP. Mean lizard masses did not differ among treatments. The mass for the lizards was 18.83±0.43 g (controls), 18.71±0.40 g (oral), and 19.19±0.42 g (dermal). The average mass-based dose for the three phthalates was approximately 435 µg/g for DMP, 478 µg/g DIBP, and 559 µg/g DNOP.

### Phthalate Oral and Dermal Exposures

Lizards were exposed to all three phthalates concomitantly, with each phthalate applied individually (i.e., 3 total doses, one each of each phthalate). Lizards exposed to phthalates were assigned to one of 4 treatment groups (n = 12 each): 24 hour oral exposure, 48 hour oral exposure, 24 hour dermal exposure, and 48 hour dermal exposure. We also had two control groups (n = 6 each), a 24 hour water gavage and 48 hour water gavage.

Oral exposure was conducted as a pseudo-gavage. The method has been described previously [8], [10]. Rather than intubate the lizards, repeatable and accurate dosing can be achieved with a micropipette and the technique is presumably less stressful for the animal. We used an Eppendorf micropipette with a volume range of 2–20 µL. The oral dosing method requires two researchers, one to firmly hold the lizard and to pull gently but firmly on the dewlap to slowly open the lizard’s mouth. The second researcher then places the pipette tip towards the rear of the mouth and administers the dose. When the dewlap of the lizard is released, the lizard will instantly close its mouth and swallow the given dose.

Dermal exposure was conducted using the same micropipette as oral dosing. The dose was placed on the ventral surface (belly). The location of dosing for each phthalate was randomized among 3 locations on the ventral surface: anterior, medial, or posterior ventral. All dermal doses were administered anterior to the pelvic girdle and posterior to the throat. Following dose application dermal treatment lizards were held for approximately 1 minute for the phthalate solution to dry/absorb to limit loss of the chemical to substrate.

### Euthanasia and Necropsy

Twenty-four or 48 hours after exposure, lizards were euthanized using CO_2_ exposure followed by decapitation. After decapitation, blood was collected in pre-weighed glass vials for later chemical extraction. All other tissues were stored in aluminum foil prior to extraction. Following blood collection, a large section of ventral skin was taken for analysis. The ventral skin sample encompassed the entire area that was dosed during dermal dosing. All necropsy equipment was cleaned and new surgical blades were used after removing ventral skin to prevent cross-contamination. We then collected adipose tissue and liver. All tissues and the remaining carcass were frozen at −80°C until extraction and analysis.

### Chemical Extraction and Analysis

All tissue samples were first extracted with methylene chloride (MeCl_2_). Adipose and blood samples were spiked with 10 µL of a 100 µg/mL surrogate (d4 deuterated di-ethyl phthalate). Adipose and liver samples were placed in a fume hood to dry for 24 hours. Ventral skin and blood were not dried prior to extraction. Samples were placed in glass vials with sodium sulfate (Na_2_SO_4_) to remove any remaining water and samples were ground with glass stir rods. Skin samples were not ground with Na_2_SO_4_ (MeCl_2_ only). We added 8 mL of MeCl_2_ to adipose tissue samples and 4 mL of MeCl_2_ to all other tissues. All samples were then placed on a VWR orbital shaker (VWR International) at 250 rpm for 24 hours. After agitating samples for 24 hours, an aliquot of 2 mL (adipose and liver samples) or 2–4 mL (blood and skin) of the extractant was filtered through a 0.45 µm PTFE syringe filter and concentrated to 1 mL using a nitrogen evaporator (adipose and liver samples, N-Evap 111, Organomation Associates Inc.) or a rotary evaporator (blood and skin samples, Buchi R-124 Rotavapor).

Phthalates were quantified using gas chromatography-mass spectrometry (HP 6890/5793) equipped with a DB-5 column (30 m×250 µm×0.25 µm). All quantitation was conducted in selective ion monitoring (SIM) mode using m/z 163 as the quant ion for DMP, m/z 153 for d4 di-ethyl phthalate, and m/z 149 for DIBP and DNOP. Concentrations in samples were not corrected for method recovery as differences between oral and dermal were generally slight and we were interested in relative residues rather than absolute residue concentrations. Instrumental method detection limits (MDL), the concentrations at which the root mean square signal to noise ratio (S/N)  = 6, ranged from 0.7–8 ppb depending on phthalate congener and specific tissues. The one exception was an MDL of 30 ppb for DNOP in liver samples.

We ran a calibration curve with every set of samples and accepted calibrations with a linear R^2^≥0.99. We ran standard checks and blank checks throughout sample runs during analysis. At the end of each sequence we ran replicate samples. We accepted standard checks when they were within ±20% of the response of the calibration standards. Replicate samples were accepted when the relative percent difference (RPD) was ≤20%. Phthalates were not detected in any MeCl_2_ blanks. If any quality assurance/quality control (QA/QC) samples failed, the entire set of samples was rerun with all QA/QC parameters repeated until acceptance criteria were passed. Extraction efficiency for d4-DEP was generally low but was consistent across treatments. For adipose tissue samples extraction efficiency averaged (mean ± SE) 41.4% (±1.7%) for oral treatments and 40.9% (±1.9%) for dermal treatments. Extraction efficiencies in blood samples averaged 71.5% (±2.7%) for oral treatments and 53% (±1.6%) for dermal treatments. Reporting limits (RL, based on the lowest calibration concentration) were dependent on tissue mass and averaged (± SD) 20.25 ng/g (±1.73) for adipose tissue, 167.56 (±7.31) ng/g for liver, 56.50 (±3.17) ng/g for blood, and 94.79 (±7.37) ng/g for ventral skin.

### Statistical Analysis

All statistical analyses were performed using R statistical software version 2.15.0 [19]. We used a randomized sampling approach to account for non-detections. A true non-detect was defined as falling between 0 and the MDL for each tissue. We defined samples in which a detection occurred, but below the reporting limit, as falling between the MDL and the reporting limit. We sampled randomly from a uniform distribution in the defined ranges to assign probable values to non-detects in the data set. In order to determine the effects of our given factors (time, exposure route, phthalate congener) we used linear mixed effects models using the lmer() function within the lme4 package in R. We created mixed effects models for each tissue in which we considered the effects of time, route of exposure, and phthalate congener as fixed effects. Because we measured multiple chemical residues in each lizard, we included individual lizards as a random effect in the statistical models. We determined which fixed factors significantly affected tissue concentration by comparing the resulting mixed effect models using the anova() function (Winter 2013). A “null” model was created which included all factors and then compared to the same model with a single factor removed. If the ANOVA comparison of the two models suggested significant differences, that factor was considered a significant factor. If either exposure route or phthalate congener were found to be significant, post-hoc analysis was performed using the glht() function within the multcomp package in R. We did not perform post-hoc analysis on the effect of time when it was significant as time was not a focus of these experiments. In order to assess the effect of randomization on results, we performed 100 iterations of the randomization procedure and quantified the proportion of p values<0.05. With only 2 exceptions, we had 100% agreement on interpretation of results across all 100 iterations. In 2 instances, there was 94% agreement, suggesting high confidence in the more common result. Therefore, results of only a single iteration are presented, but these are representative of the 100 iterations of the randomization procedure. Results are presented as means ± standard error of the mean. A level of p≤0.05 was considered to be statistically significant.

## Results

Phthalates were very rarely detected in control lizards. Out of 12 samples from control lizards, DMP, DIBP, or DNOP were not detected in adipose or liver tissues. In blood, DIBP was detected in 7 samples with a mean concentration of 207.7±49.74 ng/g. In skin samples, DIBP and DNOP were detected in 6 samples each with means of 188.5±18.7 ng/g and 364.4±108.3 ng/g, respectively. Because detections were relatively infrequent and of low concentration relative to treatment samples, concentrations detected in control sample tissues were not subtracted from treatment samples. In all tissues, control residue data was always significantly lower than both diet and dermal treatments (all p<0.011) and will not be discussed further in the results. A summary of 24 and 48 hour data is provided in supplementary materials (Table S1). The summary provides the mean (ng/g) ± SE for all detected samples in all tissues across treatments.

**Figure 1:**
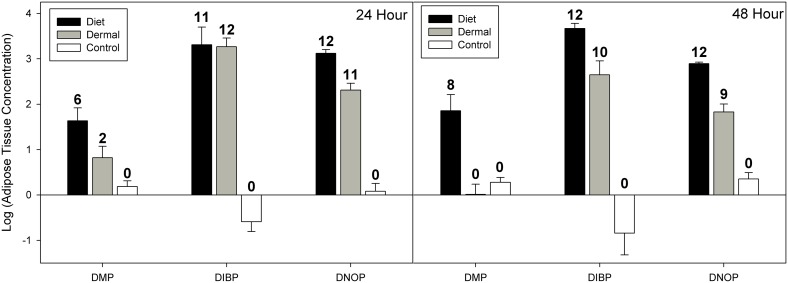
Phthalate concentrations in western fence lizard adipose tissues at 24 and 48 hour time points. Data for 24 hours and 48 hours are presented as log10 means (ng/g ± SE). Phthalate congeners are: di-methyl phthalate (DMP), di-iso-butyl phthalate (DIBP), and di-n-octyl phthalate (DNOP). Linear mixed effects models suggest a significant effect of exposure route (χ^2^ = 95.21, df = 2, p<0.001) and a significant difference between phthalate congeners (χ^2^ = 70.96, df = 2, p<0.001), but no significant effect of time (χ^2^ = 2.05, df = 2, p = 0.15). See text for complete statistical information. Numbers above error bars indicate the number of detections out of 12 samples.

For adipose tissues our mixed effects model indicated significant effects of exposure route (χ^2^ = 95.21, df = 2, p<0.001) and phthalate congener (χ^2^ = 70.96, df = 2, p<0.001), but not time (χ^2^ = 2.05, df = 1, p = 0.152; see Figure 1). Post hoc analysis of exposure routes showed that dietary exposure resulted in significantly greater residues averaged across phthalate congeners (Z = 5.98, p<0.001). For phthalate congeners, DMP was measured at significantly lower concentrations than DIBP (Z = 8.92, p<0.001) and DNOP (Z = 6.81, p<0.001). There was a significant difference between DIBP and DNOP (Z = 2.93, p<0.009) with DIBP showing slightly higher overall residues than DNOP in adipose tissue.

**Figure 2:**
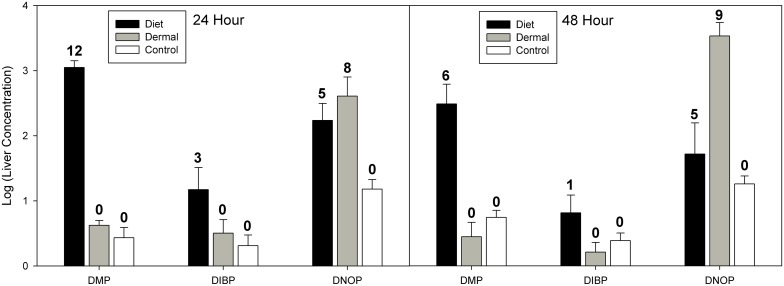
Phthalate concentrations in western fence lizard liver samples at 24 and 48 hour time points. Data for 24 hours and 48 hours are presented as log10 means (ng/g ± SE). Phthalate congeners are: di-methyl phthalate (DMP), di-iso-butyl phthalate (DIBP), and di-n-octyl phthalate (DNOP). Linear mixed effects model suggests significant effects of exposure route (χ^2^ = 31.18, df = 2, p<0.001) and phthalate congener (χ^2^ = 63.41, df = 2, p<0.001), and a marginally significant effect of time (χ^2^ = 3.58, df = 2, p = 0.059). See text for complete statistical information. Numbers above error bars indicate the number of detections out of 12 samples.

For liver, our mixed effects model indicated significant effects of exposure route (χ^2^ = 31.18, df = 2, p<0.001) and phthalate congener (χ^2^ = 63.41, df = 2, p<0.001), and no significant effect of time (χ^2^ = 2.44, df = 1, p = 0.12; see Figure 2). Post hoc analyses of exposure routes showed that dietary exposure resulted in significantly greater residues averaged across phthalate congeners (Z = 3.57, p = 0.001). For phthalate congeners, all congeners were significantly different from one another (all Z>4.3, all p<0.001). DNOP had the highest residues in liver, followed by DMP, and then DIBP.

**Figure 3:**
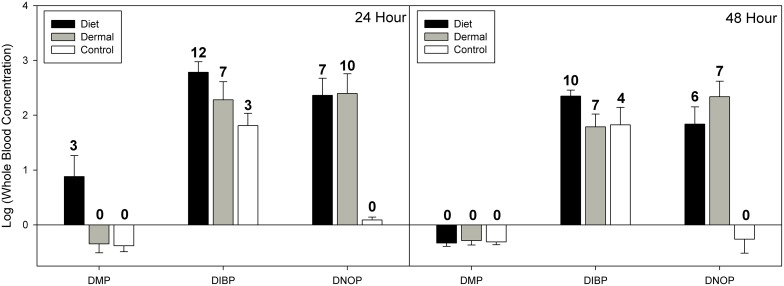
Phthalate concentrations in western fence lizard blood samples at 24 and 48 hour time points. Data for 24 hours and 48 hours are presented as log10 means (ng/g ± SE). Phthalate congeners are: di-methyl phthalate (DMP), di-iso-butyl phthalate (DIBP), and di-n-octyl phthalate (DNOP). Linear mixed effects model suggests significant effects of exposure route (χ^2^ = 31.71, df = 2, p<0.001) and phthalate congener (χ^2^ = 140.3, df = 2, p<0.001), and a significant effect of time (χ^2^ = 7.16, df = 2, p = 0.007). See text for complete statistical information. Numbers above error bars indicate the number of detections out of 12 samples. At 24 hours, 1 dermal treatment sample was lost during extraction and, therefore, detections listed are out of 11 samples.

For whole blood, when we included controls our mixed effects model indicated significant effects of exposure route (χ^2^ = 31.71, df = 2, p<0.001) and phthalate congener (χ^2^ = 140.3, df = 2, p<0.001), and a significant effect of time (χ^2^ = 7.16, df = 2, p = 0.007; see Figure 3). Post hoc analysis of exposure routes showed that oral and dermal exposure did not result in significantly different tissue residues (Z = 1.84, p = 0.157). The significant effect of exposure route in the full model is due to both dermal and dietary treatments resulting in significantly greater residues than controls. For phthalate congeners, there were significantly lower concentrations of DMP than DIBP (Z = 13.83, p<0.001) and DNOP (Z = 11.14, p<0.001). Tissue concentrations of DIBP were greater than DNOP in whole blood tissue (Z = 2.70, p = 0.020).

**Figure 4:**
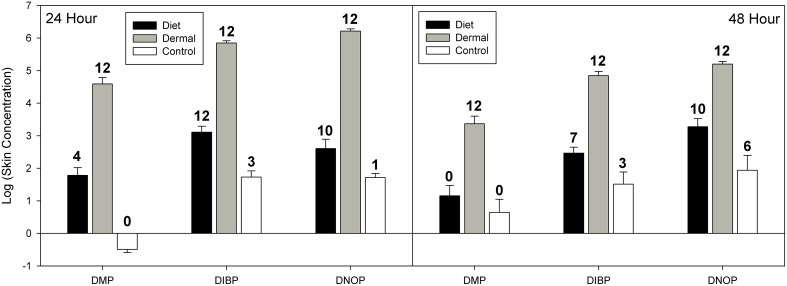
Phthalate concentrations in western fence lizard skin samples at 24 and 48 hour time points. Data for 24 hours and 48 hours are presented as log10 means (ng/g ± SE). Phthalate congeners are: di-methyl phthalate (DMP), di-iso-butyl phthalate (DIBP), and di-n-octyl phthalate (DNOP). Linear mixed effects models suggest significant effects of exposure route (χ^2^ = 122.5, df = 2, p<0.001) and phthalate congener (χ^2^ = 99.0, df = 2, p<0.001), and a significant effect of time (χ^2^ = 9.13, df = 2, p = 0.002). See text for complete statistical information. Numbers above error bars indicate the number of detections out of 12 samples. At 48 hours, 2 oral treatment samples were lost during extraction and, therefore, detections listed are out of 10 samples.

Ventral skin samples had a very different pattern than tissues discussed above (Figure 4). Mixed effects models indicated significant effects of exposure route (χ^2^ = 122.5, df = 2, p<0.001) and phthalate congener (χ^2^ = 99.0, df = 2, p<0.001), and a significant effect of time (χ^2^ = 9.13, df = 2, p = 0.002; see Figure 3). Post hoc analysis of exposure routes showed that, overall, phthalate residues were significantly greater in skin samples from dermally exposed lizards compared to those exposed orally (Z = 15.8, p<0.001). For phthalate congeners, there were significantly lower concentrations of DMP than DIBP (Z = 11.39, p<0.001) and DNOP (Z = 9.95, p<0.001). There was no significant difference between DIBP and DNOP (Z = 1.44, p = 0.321).

## Discussion

Exposure of lizards to the same quantity of phthalate via oral and dermal exposure routes resulted in greater residues for oral in some cases (adipose tissue and liver), but not blood or skin. When there were significant differences between the two exposure routes, the differences in residue concentrations were generally not large. The exception is the large difference in residue in skin samples, with dermal exposure having much greater residues than oral exposure. In addition, in liver at 48 hours, dermal exposure resulted in greater residues than oral exposure, but our statistical analysis did not allow interactive post hoc comparisons. Overall, these results suggest that similar quantities of chemicals that are delivered via oral or dermal exposure routes will result in similar uptake and subsequent body residues, at least within 48 hours. To our knowledge, this is the first study to explore the relative roles of dermal and oral exposure in contaminant uptake and distribution to tissues in any reptile species.

With few exceptions, adipose tissues had the highest number of detections. Concentrations of DIBP were generally higher than DNOP across treatments. One might expect that adipose concentrations should increase with increasing log K_ow_. However, contaminants that have very high log K_ow_ coefficients may not follow intuitive patterns. For example, in aquatic systems, very hydrophobic chemicals (e.g., log K_ow_>6) may not bioaccumulate as expected [20] compared to moderately lipophilic contaminants. In aqueous systems, it has been found that absorption across gill membranes is related to log K_ow_ in the range of log K_ow_ of 1–3, but beyond log K_ow_ = 3 chemicals appear to remain at the same bioaccumulation rate as lower log K_ow_ values [21]. Highly lipophilic contaminants (e.g., mirex, log K_ow_>7) had decreased absorption due to the extreme lipophilic nature of the chemicals. Our current results are not consistent enough to state that chemical movement across the skin is in agreement with the previous publications but it is suggestive of such a relationship. More research with a wider range of log K_ow_ values would be needed.

Liver samples had generally low detection rates across phthalate congeners. Low detections of phthalates in the liver may be related to the activity of metabolic enzymes which are high in the liver. Following oral exposure to radiolabeled phthalates, the highest proportions of residues were found in the gastro-intestinal tract, bile, and liver of various mammals (e.g., [22], [23]). After 4 days, a majority of the radioactivity was found in the urine and feces, suggesting that the previous tissues were primarily responsible for metabolic breakdown. We were unable to test for metabolic breakdown products of phthalates which may have been present in our samples.

Blood samples had more detections than liver samples. Parent compounds in blood samples up to 48 hours after exposure suggests relatively recent absorption of the phthalates from either skin or the gastrointestinal tract. Perhaps the slower reptile ectothermic physiology allowed the phthalates to continue to circulate in the blood stream even up to 48 hours after exposure. There was no significant difference in phthalate residues between oral and dermal exposures in whole blood, despite significant differences in adipose and liver tissue. Our results suggest that both exposure routes result in similar levels of circulation in blood. Perhaps the significant differences in liver are due to the fact that oral exposures are routed to the liver prior to circulation, while dermal exposure can immediately enter circulation.

Concentrations of all three phthalates were very high in extracts of ventral skin samples from dermal exposure. It is expected that a great deal of the initial dermally administered quantity would still be contained on the outside of the skin or within the skin matrix. This result suggests that a dose adsorbed/absorbed to skin may be available for uptake for several days following exposure [17]. Dietary exposure, in contrast, is usually only available for uptake from the gastrointestinal track for 24–48 hours before excretion. Surprisingly, concentrations of phthalates were detected in ventral skin samples from oral treatments as well. Detections were highest for the more lipophilic phthalates (DIBP and DNOP). Skin has previously been shown to be an excretion route for reptiles for both heavy metals [24] and lipophilic organics [25], [26]. Because a relationship between exposure level and skin concentration was found, it was suggested that shed skins may provide a minimally invasive technique to determine exposure in wild snakes at contaminated sites. Although the skins examined in our study were not shed skins, it is likely that much of the residue in the skin would be lost during the shed cycle. Our results suggest that using shed skins for biomonitoring may represent both dermal exposure that was not absorbed into the body and excretion from dietary exposure. Separating the two exposure routes would be difficult for shed skins found in the wild as a proxy for dietary exposure.

Generally, phthalate tissue residues significantly differed among congeners. In general, DMP had lower tissue concentrations than DIBP and DNOP, except in liver. This result seems fairly consistent across exposure routes suggesting that DMP is not absorbed as well as the more lipophilic phthalates, regardless of exposure route. The difference between DIBP and DNOP is more complex and is tissue dependent. DNOP concentrations were significantly greater than DIBP in liver, the reverse was found for adipose tissue. As stated earlier, very lipophilic contaminants, such as DNOP, may not behave as expected [20]. In this case, perhaps DNOP was shunted to the liver following absorption, rather than accumulating in the adipose tissue. A consistent finding was the number of detections of DMP was almost always higher in oral exposures than in dermal exposures, suggesting that an even greater lipophilicity is needed to be absorbed dermally compared to oral exposure [27]. There may be a threshold lipophilicity below which chemicals are not absorbed well across the skin.

While there has been an increase in the availability of reptile toxicity data (LC_50_s, NOAELs, etc.) there are few, if any, studies that have investigated or estimated exposure in reptiles (but see [3]). Specifically with regard to dermal exposure, no actual exposure studies are available although several instances of dermal toxicity [28]–[30] clearly point to the potential role dermal exposure plays in manifestation of toxic effects. Toxicity infers exposure as toxicity indicates a certain proportion of the chemical has crossed the skin barrier and has been transported to a site of action. Brooks et al. [28] exposed brown tree snakes to a variety of contaminants via both oral and dermal exposure routes. Oral exposure usually caused toxicity at lower doses than dermal exposure but estimated LD_50_s from both routes were always within an order of magnitude of each other. Our results comport with the results of Brooks et al. [28]. In general, oral exposure resulted in similar body residues as dermal, but in some cases oral exposure resulted in greater body residues or detections (e.g., most DMP residues, overall residues in adipose and liver tissue). Other authors have reported significant reptile mortality following dermal exposure to pesticides [28]–[31]. However, these previous reports did not quantify internal body residues, so they do not provide insight into toxicokinetic differences between oral and dermal contaminant exposure.

Although ERAs for terrestrial wildlife generally focus on estimating exposure via the dietary route, there has been some interest in exploring the role of dermal exposure but the focus has been more on avian than reptilian species. For example, following an experimental organophosphate pesticide spray, dermal exposure resulted in greater cholinesterase suppression than either inhalation or oral exposure in bobwhite quail (*Colinus virginianus*) from 8 to 48 hours post-spray [16]. Domestic pigeons (*Columba livia*) exposed to three organophosphate pesticides via oral and dermal (feet application) exposure showed responses similar to those reported by Brooks et al. [28]. Pigeons exposed orally had greater mortality, but dermal exposure resulted in longer cholinesterase inhibition [32]. This pattern may be occurring in our current results as well. The concentration of DNOP in liver samples at 48 hours appears to be much higher from dermal exposure compared to oral (our statistical methods did not allow interactive post hoc comparisons, Figure 2). This is perhaps the result of continuing exposure to DNOP from external skin residues that are still being absorbed into circulation. Finally, birds inhabiting an orchard sprayed with azinphos-methyl had detectable concentrations on skin, feathers, and feet for up to 7 days post-spray [17]. Despite the evidence to suggest that dermal exposure is important for birds, regulatory assessments continue to emphasize dietary exposure over other exposure routes [15]. Dermal exposure is ignored for several reasons. First, oral exposure is often overestimated (e.g., assuming a bird feeds only in a pesticide treated field), and it is considered that the dermal exposure is taken into consideration by overestimating oral exposure. Second, there are less data and fewer established models available for dermal exposure, likely because it is often ignored in risk assessments. This results in risk assessments that may be overly conservative and with a great deal of uncertainty with regards to exposure modeling. Improving our understanding of dermal exposure will help to improve models that can be used to estimate dermal exposure and risk.

## Conclusions

Our findings suggest that given similar doses, dermal and oral exposure to phthalates will result in relatively similar body residues, with oral exposure having generally greater residues. Chemical properties play an important role in understanding the relative importance of oral and dermal exposure. For example, DMP (lower lipophilicity) was not well absorbed in either treatment in comparison to higher log K_ow_ chemicals, but was better absorbed in oral treatments than dermal. Understanding the relationship between chemical properties and exposure routes will lead the way toward developing more accurate exposure models. In relation to ecological risk assessment, dermal exposure should not be ignored unless mathematical exposure models suggest that daily exposure from contaminated diet will greatly exceed dermal exposure. This research points to the need for better dermal exposure models to provide realistic dermal exposure estimates to compare to dietary exposures. Interest in including reptiles in the ecological risk assessment process is increasing [6]. However, the history of risk assessment is based on knowledge of bird and mammal physiology and behavior. More research is needed to understand how the exposure scenarios of terrestrial reptiles differ from birds and mammals to accurately estimate exposure for these taxa. This research represents a first step in improving our understanding of contaminant exposure in reptiles.

## Supporting Information

Table S1 **Summary of phthalate residues (mean ng/g+SE, n = number of detections) in samples with detections.** For n<3, no SE is provided. For n = 1, the value provided is the only detection.
(XLSX)
